# Effects of Salt and Nitrogen Treatments on End Use Quality in Different End Use Types of Wheat

**DOI:** 10.3390/plants14091300

**Published:** 2025-04-25

**Authors:** Jennifer N. Bragg, Jiping Liu, Matthew J. Milner

**Affiliations:** 1 Crop Improvement and Genetics Research Unit, US Department of Agriculture, Agricultural Research Service Western Regional Research Center, Albany, CA 94710, USA; 2 Robert W. Holley Center, US Department of Agriculture, Agricultural Research Service, Cornell University, Ithaca, NY 14853, USA; jiping.liu@usda.gov; 3 Plant Breeding and Genetics Section, School of Integrative Plant Sciences, Cornell University, Ithaca, NY 14853, USA

**Keywords:** salt, sodium, nitrogen, wheat, dual stress

## Abstract

Farmers frequently rely on mineral fertilizers to increase yields, improve or sustain crop productivity, and mitigate the adverse impacts of environmental stresses, including salinity. However, improper fertilization—whether inadequate or excessive—can hinder plant growth, reduce nutritional quality, and contribute to soil degradation and environmental pollution. Understanding how different levels of nitrogen (N) fertilizers and abiotic stresses such as salt impact yields and end-use quality is important to maintain food production and ensure fair crop value. In this study, we examined four types of spring wheat to investigate the role of adequate N levels in salt tolerance and their effects on end-use quality. The findings revealed no uniform response to either low N or salt treatment regarding growth or grain characteristics. All aspects, including biomass reduction, yield response variations, and grain components such as protein content, starch, or fiber, were influenced by different abiotic stresses across the various backgrounds tested. In some cases, these stresses were additive, further reducing crop value in specific genetic backgrounds, while, in others, their effects were minor. We identified varieties that are relatively tolerant to lower N levels, maintaining both yields and biomass production, as well as varieties that are less sensitive to salt, allowing them to sustain yields and biomass production. This deeper understanding of these varieties can now be leveraged to breed for improved stress tolerance across the entire life cycle, further enhancing yields under suboptimal conditions and minimizing the effects of reduced N inputs and salt tolerance.

## 1. Introduction

Soil salinity causes significant abiotic stress on agricultural crop productivity globally, jeopardizing the potential yields of crops in salt-affected soils. Numerous factors contribute to increased soil salinity, including climatic variations, geological and hydrological conditions, human activities, and excessive irrigation [1,2]. These factors suggest that the extent of salinized land used for food production will likely increase [3,4]. With an estimated 840 million people at risk of hunger by 2030, it is imperative to develop improved crop cultivation strategies to feed growing populations, particularly on marginal soils.

Common wheat (*Triticum aestivum* L., AABBDD, 2n = 42) is a globally widespread crop, serving as a staple food for many nations and a key source of dietary protein [5,6]. However, the limited availability of arable land and adverse climate conditions pose challenges to meeting the growing demand for wheat without a new agricultural revolution. Studies have shown that increasing salinity levels significantly reduce seed production, spike development, 1000-grain weight, and economic yield in both salt-sensitive and salt-tolerant wheat varieties [7]. Many marginal saline lands, such as coastal areas, have the potential for agricultural production if salt-tolerant wheat varieties can be developed [8]. Achieving this objective requires a comprehensive understanding of how high salinity affects wheat’s metabolic, physiological, biochemical, and morphological properties and gene expression. With wheat having many end uses, from making bread, cakes, and cookies to use in animal feed, it is essential to understand how low salinity levels impact protein content and other grain and agronomic components [9,10,11]. In the US market, there are six possible types of bread wheat, with the varieties being either winter or spring planting, hard or soft flour, and red or white grain. Hard spring wheats tend to have a higher protein content and strong gluten and are often blended with other types of wheat to improve the strength of a flour blend, whereas soft spring wheats have a lower protein and less gluten strength and are typically used for cookies, crackers, pastries, flatbreads, and pretzels. The main differences between the red versus white wheats are color and flavor, with red wheats having a nuttier taste and white wheats having a sweeter flavor.

Nitrogen (N) fertilizer is one of the most essential inputs in crop production and is applied in larger quantities than any other nutrient. However, the response of plants, particularly wheat, to N fertilizer is inconsistent across genetically diverse varieties [12,13]. This variability can be due to differences in N uptake versus utilization and how plants adapt to suboptimal growing conditions [14]. These differences include genetic variation and underlying differences in ultimate end-use quality, as N is a major consideration when farmers decide what to plant. So, understanding how particular varieties respond to N can help adapt wheat to challenging climates and mitigate additional stress [15,16]. In other plant species, it has been shown that a sufficient supply of N is essential to improving salt tolerance by enhancing its osmotic balance [17].

In this study, we set out to test the role of N in salt tolerance across four types of wheat. We sought to investigate how these varieties respond to salt stress, how the interplay between N and salinity manifests in different genotypes, and whether there are shared mechanisms in how plants cope with low N and salt stress. By addressing these questions, we hope to contribute to the development of wheat varieties better suited to marginal soils and challenging environmental conditions.

## 2. Results

To understand how different types of wheat behave, we tested two hard and soft red spring wheat varieties (Butte, Bobwhite) and two hard and soft white spring wheat varieties (Otis, Fielder). These varieties represent diverse US wheat pedigrees. They were grown under low and high N conditions, with and without salt stress (electrical conductivity, EC ~ 4.2). We aimed to evaluate their responses to limited N, excess Na, and the combination of these stresses.

**Figure 1:**
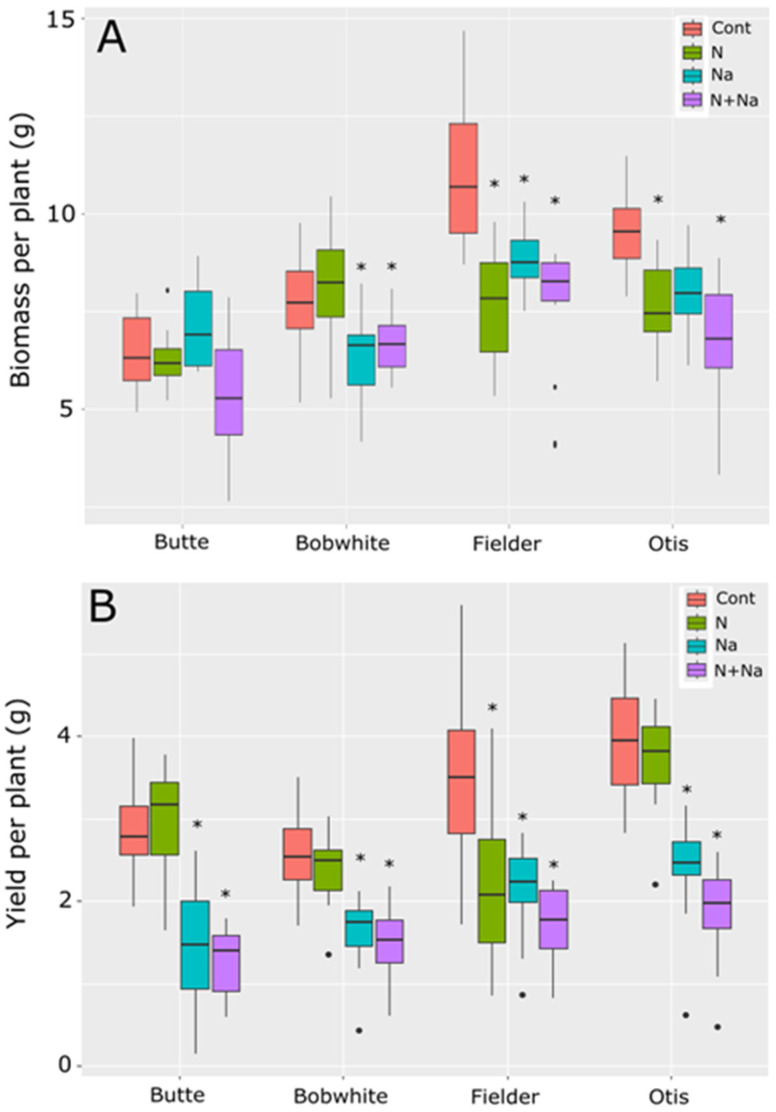
Biomass and yield of four spring wheats grown under nitrogen and salt stress conditions. (**A**) Biomass produced per plant. (**B**) Yield produced per plant. Cont = high N without salt, N = low N without salt, Na = high N with salt, N+Na = low N with salt. Star indicates significant difference of the treatment relative to the control (*p* val. < 0.05).

Overall biomass production varied significantly among the four varieties under at least one imposed stress (Figure 1A). Each stress—low N or salt—significantly reduced biomass production compared to the control treatment (*p* < 0.001). Total biomass production varied according to the line tested (Figure 1A). Butte maintained its biomass production under both low N and salt stresses, while Bobwhite showed a significant reduction in biomass production under salt stress, regardless of the amount of N supplied. Fielder and Otis were highly sensitive to individual stresses, but the combined stress did not further suppress biomass production (*p* < 0.05).

Yield responses did not mirror biomass production trends (Figure 1B). In Butte and Otis, no significant difference in yield was observed between the high and low nitrogen treatments. Still, a significant fall in yield was observed when both were grown under salt stress, and the combined salt and low-N stress further reduced yield compared to all other treatments (*p* = 0.02 and *p* < 0.001, respectively). Bobwhite did not respond to the N treatments but showed a significant yield reduction under salt stress alone and the combined stress (*p* < 0.001). Fielder was highly susceptible to either low N or salt stress, but the combined stress did not have an additional detrimental effect on yields. Fielder generally produced the highest biomass, but this was not translated into yield. Otis and Butte had the best nitrogen use efficiency (NUE), defined as grain produced per unit of N, maintaining yields under lower levels of N.

**Figure 2:**
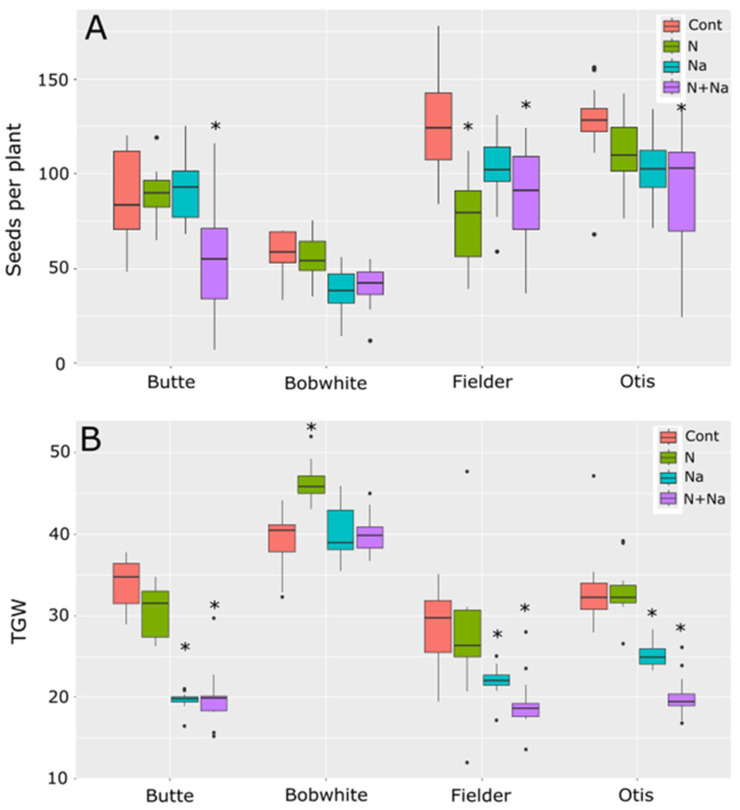
Seed production and thousand-grain weight (TGW) of four spring wheats grown under various nitrogen and salt stress conditions. (**A**) Number of seeds produced per plant. (**B**) TGW of seeds grown under various abiotic conditions. Star indicates significant difference of the treatment relative to the control (*p* val. < 0.05).

The main drivers of yield differences were also different between the lines tested (Figure 2). The Butte’s yield loss was primarily due to reduced thousand-grain weight (TGW), with no significant differences in seed number per plant. For Bobwhite, while the change in seed number was insignificant for either stress or combinational stress, only one of the treatments showed a difference in TGW, suggesting smaller differences in seed number and grain weight combined to lower yields when salt stress was present. The sensitivity observed in the variety Fielder was due to a change in seed number in response to N stress alone and a lower TGW for salt stress; both stresses combined caused a lower TGW and seed number. Finally, Otis was sensitive mainly to TGW and, to a lesser extent, the seed number, although only the dual stress showed significantly lower seed numbers than the control (*p* < 0.001). No single stress showed a significant deviation from control conditions in seed metrics for Otis (*p* = 0.89 and 0.13 for N and salt, respectively).

**Figure 3:**
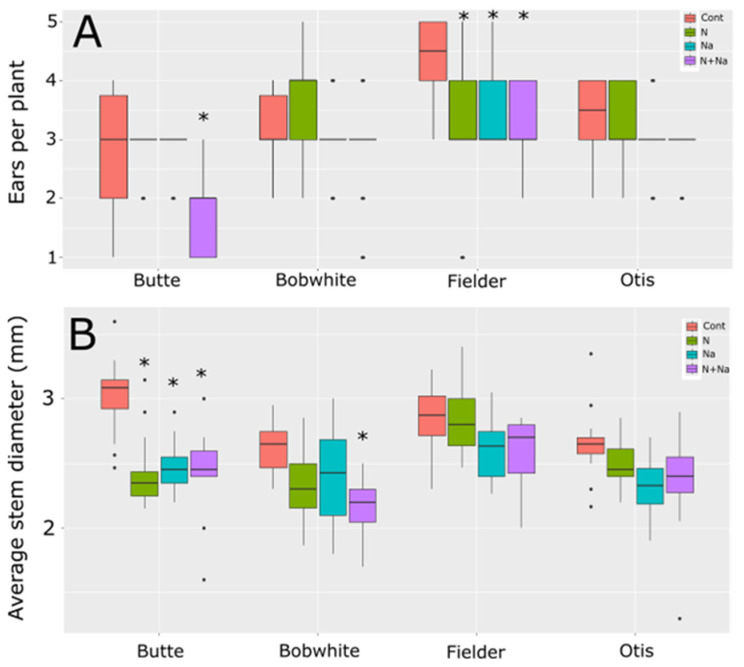
Tiller number and stem diameter of wheat grown under different nitrogen and salt stresses. (**A**) Ears per plant. (**B**) Average stem diameter of the third node at maturity. Star indicates significant difference of the treatment relative to the control, with a *p* val. of less than 0.05.

We also measured stem diameter and tiller number to understand other potential drawbacks related to low N or salt stress (Figure 3). Only two lines showed differences in the number of ears produced per plant. Butte showed significantly lower ear production when grown under both low N and salt conditions; otherwise, it maintained an average of three ears per plant for the other three growth conditions. Fielder, by comparison, showed reduced ears per plant under any form of stress, whether it was a single stress or a combination of salt and low N. Bobwhite and Otis showed no change in ear number in response to either type of stress or their combination. When assessing growth via stem diameter, the Butte variety exhibited pronounced sensitivity to any single stress: low N reduced stem diameter by nearly 20%, while salt stress caused an 18% decrease (Figure 3B). Apart from Butte, Bobwhite showed a significant reduction in stem diameter under dual stress, with an average decrease of over 18% compared to control conditions. Fielder and Otis did not change their stem diameters in response to any treatment or the combination of stresses.

**Figure 4:**
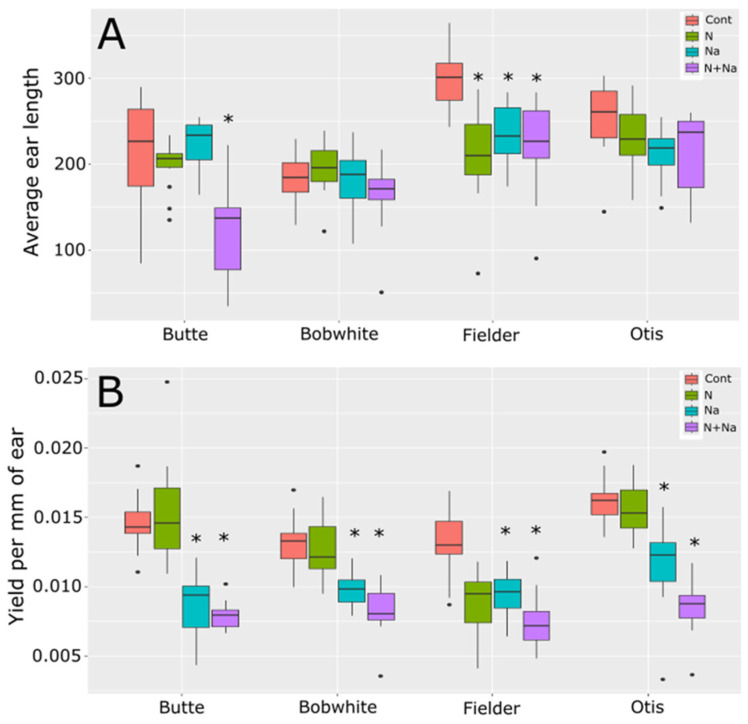
Ear length and seed set per mm of ear length. (**A**) Average total ear length per plant. (**B**) Yield per mm of ear length (yield per plant/total ear length per plant). Star indicates significant difference of the treatment relative to the control, with a *p* val. of less than 0.05.

To understand how the treatments affected fertility, we also measured ear length and yield per mm of ear length (Figure 4). Average ear length was determined by calculating the total length of all ears produced per plant. Significant differences in ear length were observed in only two of the four varieties tested when the plants were stressed. Butte showed significantly reduced ear length under dual stress and Fielder showed significantly shorter ear lengths under both the single and dual stress conditions. This change in ear length suggests that salt stress negatively affected ear fertility as all lines showed reduced yield per mm of ear length (Figure 4B). This reduced yield was most likely driven by the reduced TGW observed under the same growth conditions, as shown in Figure 2B for three of the four varieties tested, rather than being attributable primarily to pollen fertility.

**Figure 5:**
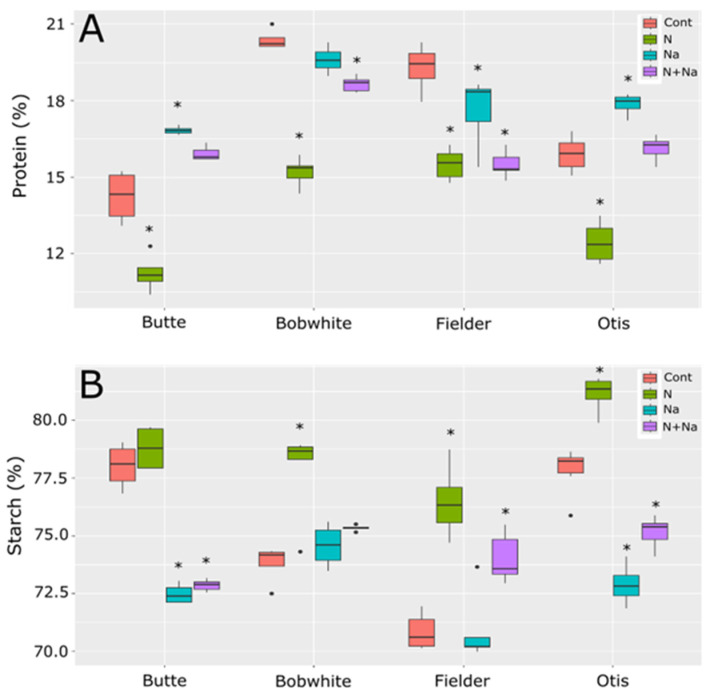
Protein and starch levels in grains of four end use types of spring wheat. (**A**) Protein levels in mature grains. (**B**) Starch levels in the mature grains. Star indicates significant difference of the treatment relative to the control, with a *p* val. of less than 0.05.

Protein and starch levels in the lines tested also showed significant differences in their overall levels as well as in their responses to the various stresses imposed (Figure 5). For protein content, a consistent increase in protein content with salt stress was not seen, as previous reports suggested [1,2]. With Butte and Otis showing increased protein content under salt stress, Bobwhite showed no change in protein content and Fielder showed a significantly lower protein level than control conditions. A consistent decrease in protein content related to N levels was observed and, even when salt stress did increase, the combination of salt and low N levels always resulted in a significantly lowered protein content compared to salt treatment alone. Surprisingly, the starch content did not mirror the protein concentration trends. Butte showed much lower starch content when salt was present, regardless of N treatment, but N levels did not appear to influence starch levels. By comparison, Bobwhite showed higher starch levels under low N levels, but the addition of salt did not increase starch levels in either high or low N conditions. Finally, Fielder and Otis showed similar patterns, with low N leading to increased starch levels in their grains. Salt levels lowered starch levels significantly for Otis but not Fielder and the dual stress showed an intermediate level compared to control conditions or low N.

**Figure 6:**
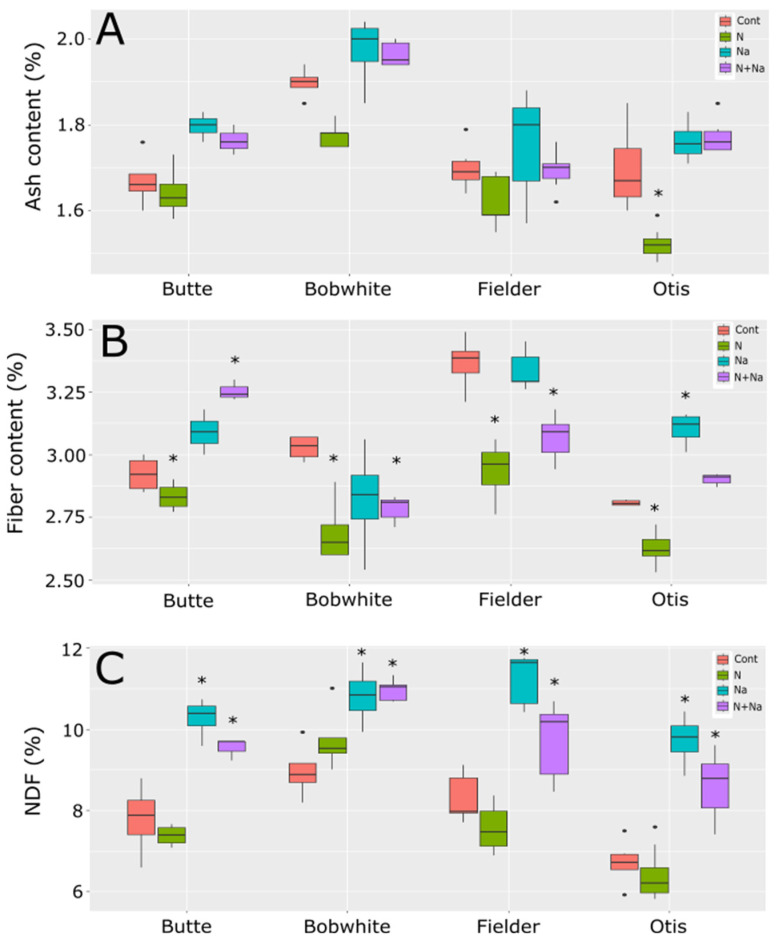
Ash, fiber, and NDF levels in grains of four end use types of spring wheat. (**A**) Ash content in mature grains under various abiotic conditions. (**B**) Fiber levels in the mature grains grown under various abiotic stresses. (**C**) NDF levels in the mature grains of wheat grown under four different abiotic conditions. Star indicates significant difference of the treatment relative to the control, with a *p* val. of less than 0.05.

Ash levels mostly did not change across the treatments in all tested lines, except for Otis, which exhibited a significant reduction in ash content in the grain under low N conditions (Figure 6). Fiber levels fluctuated with most stresses, but these changes were not consistently predictable. All lines showed significantly lower fiber levels under low N conditions. Generally, salt did not affect fiber levels, except in Otis, where higher fiber levels were observed with added salt. The combination of low N and salt significantly lowered fiber levels in Bobwhite and Fielder but resulted in significantly raised fiber levels in Butte. Finally, neutral detergent (NDF) showed a far more predictable response to salt treatment, significantly increasing NDF levels in the grain, regardless of nitrogen status. Low N alone did not affect NDF levels in any of the lines tested.

**Figure 7:**
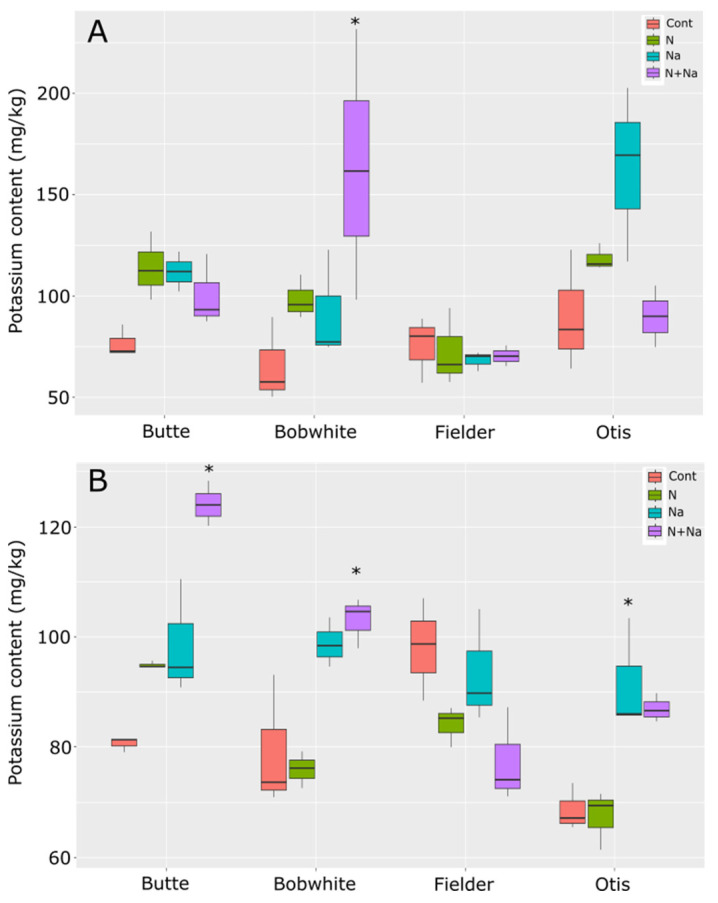
Potassium content in leaves and seeds of mature wheat plants grown under various abiotic stresses. (**A**) Leaf potassium content. (**B**) Seed potassium content. Star indicates significant difference of the treatment relative to the control, with a *p* val. of less than 0.05.

Potassium concentrations generally remained stable across the various stress treatments. Overall, the treatments that changed potassium levels in the leaves were the salt versus control conditions as well as the dual stress compared to control conditions (*p* = 0.039 and 0.021, respectively). Although significant differences in K levels were observed among all lines except Fielder in either leaves or seeds, the only significant difference seen in the K content of the leaves of a particular line was in the dual stress condition of Bobwhite relative to its control (*p* = 0.003) (Figure 7). The other lines tested did not show significant differences in leaf K content across treatments. This pattern mirrored the ash content data, suggesting these lines did not differ in their response to either stress. In the seeds, the addition of salt or dual stress resulted in significant differences compared to the control conditions. Notably, the dual stress treatment increased potassium levels in the grains of Butte and Bobwhite, while salt-only stress raised potassium levels in Otis grains. However, this trait also showed some variation, as seeds from Fielder grown under the dual stress were marginally non-significant compared to control conditions (*p* = 0.056), but there was a lower K concentration compared to the other lines, which all showed increased K levels.

**Figure 8:**
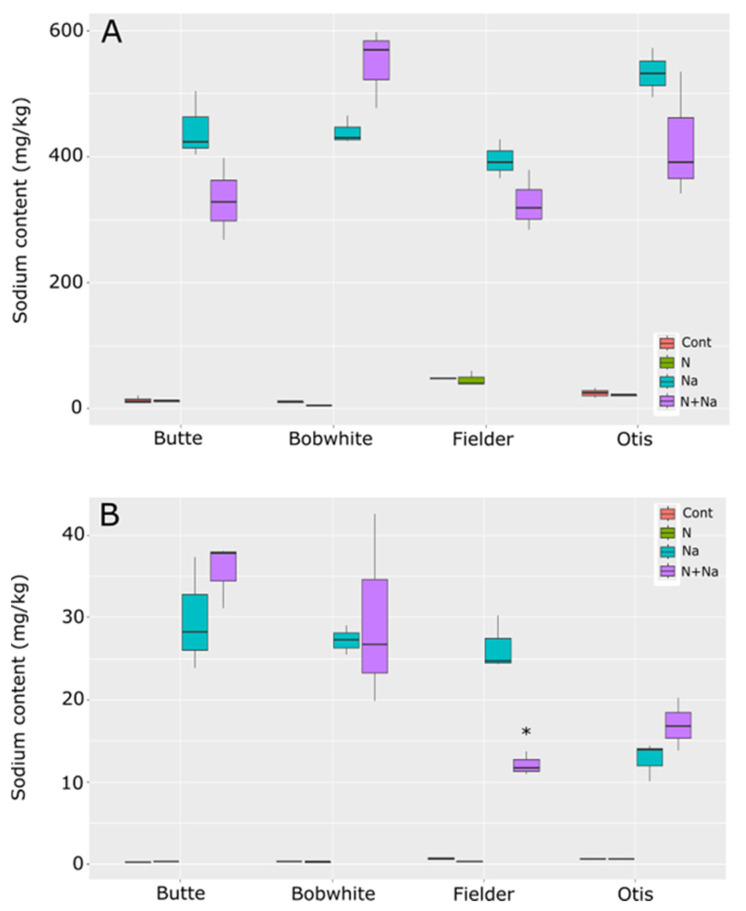
Sodium content in leaves and seeds of mature wheat plants grown under nitrogen and salt stresses. (**A**) Leaf sodium content. (**B**) Seed sodium content. Star indicates significant difference of the treatment relative to the salt treatment, with a *p* val. of less than 0.05.

Sodium concentrations differed by line and treatments in the leaves, with all lines showing significantly higher Na concentrations in the leaves than in the control conditions. The comparisons between salt and dual stress conditions were more relevant (Figure 8). Only Fielder and Butte showed similar levels of Na in the leaves, as did Otis and Bobwhite, with the reciprocal comparisons showing significantly different leaf sodium levels between the groups. Overall, no significant variation was seen between treatments within any specific line. In the seeds, the dual stress treatment had a significant impact only on Fielder, where the combination of low N and salt resulted in significantly lower salt levels in the grains than salt treatment alone. Otherwise, no effects of N were seen on sodium levels in the grains across the tested lines.

## 3. Discussion

Understanding how lines respond to changes in agronomic practices is key to increasing and maintaining crop yields under stressed conditions. To understand how small genetic differences influence end uses of wheat, we grew four lines spanning four of the six US wheat classes under two different abiotic stresses (low N and salt stress), along with their dual combination, to assess their effects on growth and yield-related traits. The findings revealed that most traits measured in these experiments lacked consistent trends across the lines tested, indicating that various factors can influence ultimate yield and relative tolerance. This work highlights that traits associated with maintaining or improving crop yields—such as stress tolerance and growth efficiency—operate through complex and multifactorial mechanisms rather than simple pathways. This work also highlights some of the key limitations in improving salt tolerance in wheat, as previous experiments often subjected plants to high salt levels to assess their tolerance [7,8]. Recent extensive screenings of early seedling tolerance to low N or salt have employed extreme stress levels to evaluate overall tolerance or toxicity responses. These screenings are conducted at the early stage, where differences in biomass production—a poor proxy for final yield—are measured. Alternatively, they may focus narrowly on yield metrics (a specific biomass component) while overlooking other stress-adaptive traits critical to agricultural resilience. Salinity tolerance and N deficiency resilience are polygenic and highly complex traits, making understanding tolerance mechanisms across different developmental stages essential to optimize agronomic performance [6]. While our data revealed a strong correlation between total biomass and final yield, deep analysis exposed cultivar- and treatment-specific disparities. These variations enabled us to disentangle stress-induced phenotypic responses (e.g., yield loss) from their genetic or physiological drivers. Salt generally caused greater reductions in both yield and biomass compared to low N. However, Butte and Otis exhibited less pronounced declines under low N stress than Fielder and Bobwhite. This pattern suggests that Butte and Otis possess greater NUE or compensatory mechanisms under N limitation, reflecting genetic divergence in nutrient utilization strategies.

To understand how the various abiotic stresses impacted overall yields, we investigated how the essential and toxic levels of abiotic stress differed in wheat. It was observed that salt stress alone had no significant effect on seed number per plant. In contrast, low N significantly reduced the seed set in Fielder and three of the four lines under dual stress, suggesting that N availability plays a dominant role in reproductive success compared to salinity. Conversely, TGW declined under salt and dual stress, whereas low N showed no effect on TGW, which even increased in Bobwhite. This divergence led to significant differences in the yield per mm of ear produced, underscoring seed weight as a critical factor in salt stress tolerance (Figure 4). Notably, multiple genes regulating TGW have been found in wheat, suggesting that targeting natural or engineered variations in seed size-related genes/loci could improve yield stability under salt stress [18,19,20,21,22,23].

The stresses also influenced the ears per plant and stem diameter, key determinants of yield potential under field conditions. Mostly, the ear number remained constant across treatments, with two exceptions: Butte exhibited reduced ear number under dual stress, while Fielder showed declines under all imposed stress conditions (Figure 3). This trend mirrored the overall ear length data: Butte’s ear length decreased significantly only under the dual stress, whereas Fielder’s declined under all stresses imposed (Figure 4).

Stem diameter responses varied by cultivar. Butte displayed heightened sensitivity to all stress treatments, with low N and salt stress reducing stem diameter by ~20% and 18%, respectively, a trait linked to increased lodging risk. Bobwhite also showed reduced stem diameter under salt stress (18% decrease), while Fielder and Otis maintained stable stem diameters across treatments. These cultivar-specific differences highlight how genetic background modulates structural resilience to abiotic stress.

Grain composition also showed important differences as salt stress has been shown to increase the protein content of grain and lower N fertilization has been shown to reduce protein content [3,14,24]. While we saw a consistent reduction in protein content across all lines under low N, only two of the four lines tested showed increased protein content in the grain under salt stress. This reduction of protein content was generally associated with increased starch content in three of the four lines tested. However, the inverse relationship was not absolute (e.g., the highest starch content did not correspond to the lowest protein content by comparison), suggesting independent regulatory pathways for these components. This finding highlights the potential to modify the protein and starch content of the grains independently.

Fiber content responded divergently to stress treatments: low N reduced the fiber content in all lines tested, aligning with earlier singular-variety reports [15], whereas salt stress had no effect on the fiber in three cultivars but increased it in Otis. Under combined stress, fiber trends were less consistent: Butte showed elevated fiber content, while Bobwhite and Fielder exhibited declines, despite all three cultivars displaying reduced fiber under low N alone. Neutral detergent fiber (NDF) in grains, a critical metric for feed quality, rose significantly in all cultivars under salt-containing treatments (Figure 6). This increased NDF could compromise palatability and digestibility in animal feed, potentially limiting intake [25]. These results underscore the complexity of stress-induced compositional changes, where genetic background modulates responses to individual or combined stressors. The decoupled starch–protein relationship and cultivar-specific fiber dynamics highlight opportunities to optimize both nutritional and agronomic traits through targeted breeding.

Finally, potassium and sodium levels were measured in the leaves and grain to understand how sodium moved through the plant. No consistent trends emerged across the lines tested, suggesting that differences in K/Na homeostasis were not the primary drivers of the phenotypic differences observed in other traits (e.g., yield, biomass). Maintaining high K retention and minimizing Na accumulation in shoots—a hallmark of salt tolerance linked to transporters like HKT1—remains a central focus in the literature [26,27,28,29]. Indeed, recent advances in understanding genetic diversity—particularly the role of genes such as *HKT1* in maintaining low Na^+^ levels in above-ground tissues—have demonstrated their importance in enhancing salt-stress tolerance [30]. However, our data suggest that these mechanisms alone do not fully explain cultivar-specific stress responses.

This finding aligns with emerging evidence of genetic complexity: for instance, Wang et al. (2024) recently showed that only a small subset of 300 tested wheat lines carried an indel in the *SPL6* coding region, which is a gene responsible for inhibiting the expression of the potassium transporter *TaHKT1;5-D* [30]. *TaSPL6-D^In^*, the allele with a truncated predicted protein, could not inhibit the expression of *TaHKT1;5-D* by binding its promoter and increasing salt tolerance [30]. This example of a more complex regulation of activity and additional transcription factors could also regulate multiple loci. Such findings underscore that salt tolerance likely arises from diverse genetic networks, where both canonical genes (e.g., *HKT1*) and their regulators fine-tune the stress responses and adaptation.

Overall, this study provided limited evidence to support nitrogen’s role in increased salt tolerance, in contrast to observations in other species. However, several traits measured did show altered phenotypes under combined salt and low N stress, suggesting synergistic or antagonistic interactions between these stressors. A key unresolved question is whether the form of N fertilization rather than its quantity could modulate salt tolerance. For instance, substituting ammonium nitrate (commonly used in the US) with potassium nitrate might improve salt tolerance by leveraging potassium’s ability to antagonize sodium uptake at root surfaces. While such N-form-dependent tolerance mechanisms have been documented in other species [31,32,33], their applicability to wheat remains untested. Further studies should explore how N source, dose, and timing shape plant responses to combinational abiotic stresses, particularly in genetically diverse cultivars. Also, since two of the lines tested are easily transformable, with one being fully sequenced, understanding the genes involved in the differences observed may also show how underlying genetic differences play a role in improving stress tolerance to N and salt stress.

## 4. Materials and Methods

Plant Growth: Wheat plants were grown in a randomized block design in Deepot stands (Stuewe & Sons) filled with cells 2″ wide and 10″ long, with an internal volume of 410 mL. The experiment was conducted in a glasshouse set to day–night temperatures of 20 °C and 15 °C, respectively. Each cell was filled with Sunshine Mix soil and seeds were germinated directly in the soil. Four wheat varieties—Fielder (accession number: CItr 17268), Bobwhite (accession number: PI 519665), Otis (accession number: PI 634866), and Butte (accession number: CItr 17681)—were selected for comparison. Fifteen plants of each variety were grown under low and high N with and without salt stress for each treatment.

Plants were grown to Zadok stage 13, at which point, the first dose of N was applied. The second dose for the high N treatment was given around Zadok stage 31 and the final dose was applied during Zadok stage 50. Each N dose consisted of 800 mg of NH_4_NO_3_ per Deepot, delivered in a 10 mL volume. Salt stress was imposed by adding 20 L of a 200 mM NaCl solution, maintaining a measured electrical conductivity (EC) of approximately 4.2 for the rest of the plant’s life cycle. The salt solution was applied in a water basin covering the lower third of the Deepot.

Agronomic Measurements: Ear length and stem diameter were measured using a digital caliper before harvest on fully mature plants for each ear and stem that produced grain. At maturity, total shoot dry weight, seed weight (yield per plant), seed number, seed size, and tiller number (defined as ears with seeds) were recorded after the plants were dried for two weeks at 35 °C. Grain quality parameters, including protein, ash, fiber, NDF, and starch content, were analyzed using near-infrared spectroscopy (NIR) (Perten Instruments model DA7250).

Mineral Analysis: Mineral contents were determined for leaf and seed samples. Samples were treated with 3.0 mL of a 60:40 HNO_3_ and HClO_4_ mixture in a Pyrex glass tube and left overnight to destroy organic matter. The mixture was then slowly heated to 120 °C for two hours in a heating block. The temperature of the heating block was then raised to 145 C for two hours. If necessary, more nitric acid (1–2 mL) was added to destroy the brownish color of the organic matter. Then, the temperature of the heating block was raised to 190 C for ten minutes. The cooled samples in the tubes were then diluted to 10 dH_2_0, vortexed, and transferred into auto sampler tubes to run in ICP-AES. Appropriate standards were prepared in 2% perchloric acid and the elements of interest were measured using Thermo iCAP 7500.

Statistical Analysis: Two-way analysis of variance (ANOVA) was conducted to assess significance between the tested varieties using the aov() function and TukeyHSD commands in R. A least significant difference (LSD) at a 5% probability level was used as a post-hoc test to determine significance. The results were plotted using the ggplot2 and ggpubr packages in R [34,35].

## Data Availability

The original contributions presented in this study are included in the article. Further inquiries can be directed to the corresponding author.
